# Total Synthesis and the Biological Activities of (±)-Norannuradhapurine

**DOI:** 10.3390/molecules14010089

**Published:** 2008-12-29

**Authors:** Surachai Nimgirawath, Rujee Lorpitthaya, Asawin Wanbanjob, Thongchai Taechowisan, Yue-Mao Shen

**Affiliations:** 1 Department of Chemistry, Faculty of Science, Silpakorn University, Nakorn Pathom 73000, Thailand; E-mails: G050010@ntu.edu.sg (R. L.), winsusc@gmail.com (A. W.); 2 Department of Microbiology, Faculty of Science, Silpakorn University, Nakorn Pathom 73000, Thailand; E-mail: tthongch@su.ac.th (T. T.); 3 Institute of Botany, Chinese Academy of Sciences, Kunming 650204, P. R. China. E-mail: yshen@mail.kib.ac.cn (Y-M. C.)

**Keywords:** Alkaloid, Anti-inflammatory Activity, Aporphine, Isoquinoline, Synthesis, Radical Cyclization.

## Abstract

The structure previously assigned to the phenolic noraporphine alkaloid, (-)-norannuradhapurine has been confirmed by a total synthesis of the racemic alkaloid in which the key step involved the formation of the C ring by a radical-initiated cyclization. although inactive against *Staphylococcus aureus* ATCC25932, *Escherichia coli* ATCC10536 and *Candida albicans* ATCC90028, (±)-norannuradhapurine inhibits the production of NO, PGE_2_, TNF-a, IL-1b and IL-6 and the expression of iNOS and COX-2 in RAW 264.7 macrophages stimulated with LPS *in vitro*.

## Introduction

(-)-Norannuradhapurine (**1a**) is a phenolic noraporphine alkaloid isolated for the first time from the bark and leaves of *Polyalthia acuminate* Thw. (Annonaceae) [1] and subsequently from the bark and wood of *Fissistigma glaucescens* (Hance) Merr. and the wood of *Fissistigma oldhamii* (Hemsl.) Merr. (Annonaceae) [2]. (-)-Norannuradhapurine has been shown to exhibit strong inhibition of adenosine 5’-diphosphate (ADP)-induced, collagen-induced and platelet-activating factor (1-*O*-alkyl-2-acetyl-*sn*-glycero-3-phosphocholine)-induced platelet aggregations [3]. In addition, (-)-norannuradhapurine has exhibited a broad spectrum of growth inhibitory activities against murine and human leukemic cells with IC_50_ values around 3mM and it also has strong inhibitory effects on DNA, RNA and protein biosynthesis [4]. In view of these interesting biological activities and the fact that the alkaloid occurs only in minute quantities in Nature, we decided to undertake a total synthesis of the alkaloid to establish its structure and also to make it more accessible for anti-microbial and anti-inflammatory activity studies.

## Results and Discussion

### Synthesis

**Scheme 1:**
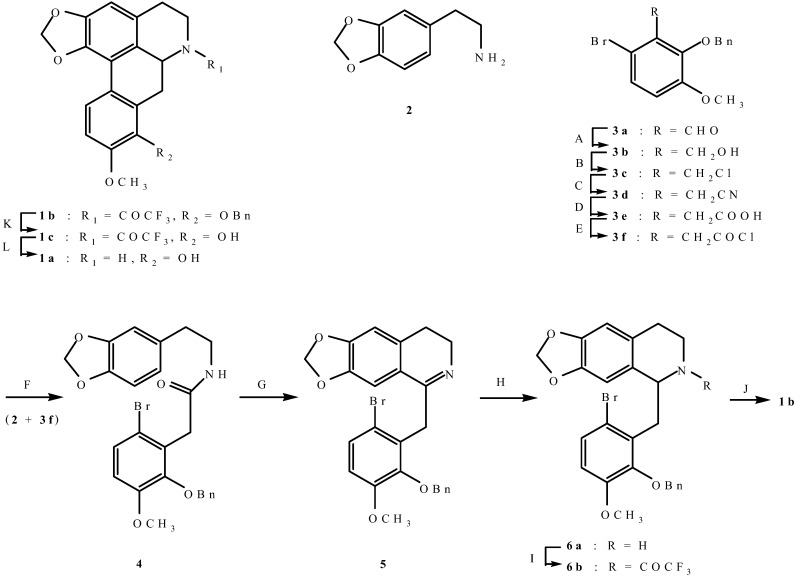
Synthetic route to (±)-norannuradhapurine (**1a**).

The strategy employed for the synthesis of (±)-norannuradhapurine (**1a**) was based on the construction of ring C by a radical-initiated cyclization which has proved successful in the syntheses of other aporphine alkaloids [5,6,7,8] (Scheme 1).

For this purpose, the required starting materials were 3,4-methylene-dioxyphenethylamine (**2**) [9] and 2-benzyloxy-6-bromo-3-methoxyphenylacetic acid (**3e**). Thus, benzylation of 6-bromo-2-hydroxy-3-methoxybenzaldehyde [10] gave **3a**, which was converted into **3b-e**, respectively by conventional methods. Condensation of 2-benzyloxy-6-bromo-3-methoxy-phenylacetyl chloride (**3f**) with 3,4-methylenedioxyphenethylamine (**2**) gave amide **4**, which was converted into **5** by a Bischler-Napieralski reaction. Reduction of **5** with sodium borohydride gave **6a** which was treated with trifluoroacetic anhydride to give **6b**. Treatment of **6b** with tributyltin hydride and 2,2'-azobis(isobutyronitrile) afforded **1b** in 40% yield. The structure of **1b** was supported by ^1^H-NMR data, in which H-11 gave rise to an unusually low-field doublet at d 7.86 and the 1,2-methylenedioxy protons gave rise to an AB quartet at d 6.02 (*J* = 1.20 Hz ), characteristic of an aporphine moiety bearing a methylenedioxy group at those positions. Attempts to remove the trifluoroacetyl group from **1b** under basic conditions were fruitless, possibly due to steric hindrance of the benzyloxy group on ring D. Fortunately, catalytic hydrogenolysis of **1b** went smoothly to give **1c**, whose trifluoroacetyl group was smoothly removed to give (±)-norannuradhapurine (**1a**), the ^1^H-NMR spectral data of which were in excellent agreement with those reported for natural (-)-norannuradhapurine [1]. Since the ^13^C-NMR spectral data of the natural alkaloid have not been previously reported, we have reported here these data from spectra measured both in CDCl_3_ and *d_6_*-DMSO for future reference.

*Reagents and conditions*: A) NaBH_4_/ethanol; B) SOCl_2_, pyridine/chloroform; C) NaCN, KI/ water-acetone; D) i. KOH/water-ethanol; ii. H_3_O^+^; E) SOCl_2_/benzene; F) 10% NaHCO_3_/ chloroform; G) POCl_3_/benzene; H) NaBH_4_/ethanol; I) (CF_3_CO)_2_O, Et_3_N/chloroform; J) Bu_3_SnH, AIBN/dry toluene; K) H_2_/Pd/C/ethanol; L) K_2_CO_3_/methanol-water.

### Biological activity

**Figure 1:**
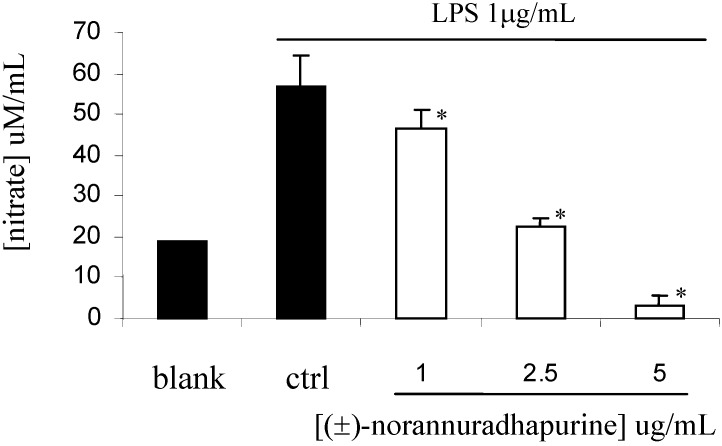
Evaluation of nitrite production by RAW 264.7 cells stimulated for 24 hours with LPS alone or combination with increasing concentrations (1-5 mg/mL) of (±)-norannuradhapurine. The values are the means of at least three determinations ± SD. Probability levels (Student’s *t*-test): * *p* < 0.05 *vs*. LPS-treated group.

**Figure 2:**
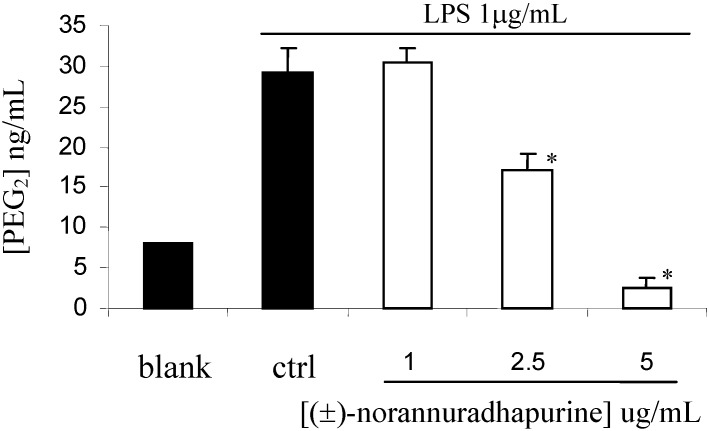
Effect of (±)-norannuradhapurine on PEG_2_ production in LPS-induced RAW 264.7 macrophage for 24 hours. The values are the means of at least three determinations ± SD. Probability level(Student’s t-test): * *p* < 0.05 vs. LPS-treated group.

**Figure 3:**
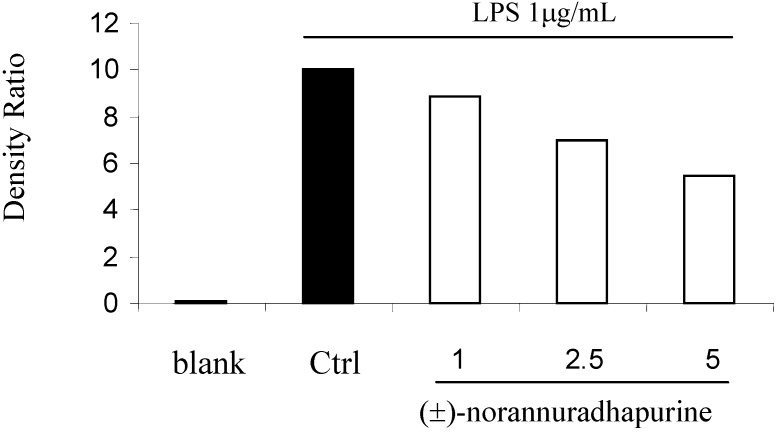
Effect of (±)-norannuradhapurine on iNOS protein production by LPS-induced RAW 264.7 macrophage for 24 hours.

**Figure 4:**
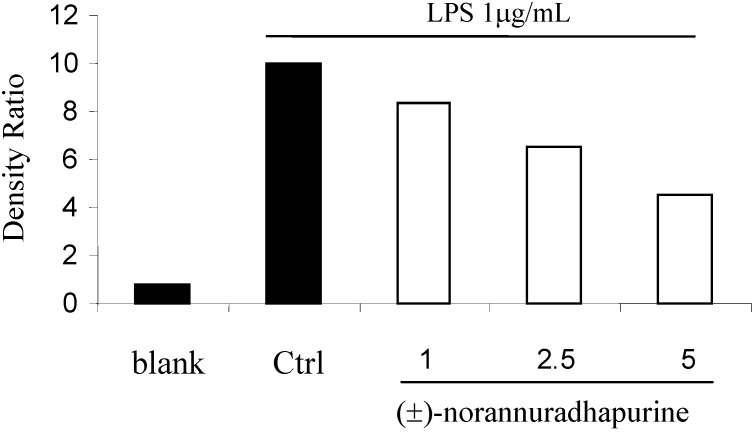
Effect of (±)-norannuradhapurine and LPS-induced COX-2 protein expression in RAW 264.7 cells.

At a concentration of 256 mg/mL, (±)-norannuradhapurine was inactive *Staphylococcus aureus* ATCC25932 *Escherichia coli* ATCC10536 and *Candida albicans* ATCC90028. In the course of our studies on its anti-inflammatory activity, we have found that (±)-norannuradhapurine inhibits NO production in murine macrophage RAW 264.7 cells stimulated with LPS (Figure 1). Next we investigated the effect of (±)-norannuradhapurine on the release of PGE_2_. Compared with the untreated control, LPS (1 mg/mL) induced a great production of PGE_2_ in RAW 264.7 cells. (±)-Norannuradhapurine (1–5 mg/mL) inhibited the production of PEG_2_ in RAW 264.7 cells stimulated with LPS in a concentration-dependent manner (Figure 2). To elucidate the mechanism of the inhibitory effect of (±)-norannuradhapurine on NO and PGE_2_ production, we investigated their effects on iNOS and COX-2 expression levels, respectively. In response to LPS, the iNOS and COX-2 induction were markedly increased, (±)-norannuradhapurine significantly decreased the iNOS and COX-2 protein expression in a concentration-dependent manner (Figure 3 and Figure 4).

**Figure 5:**
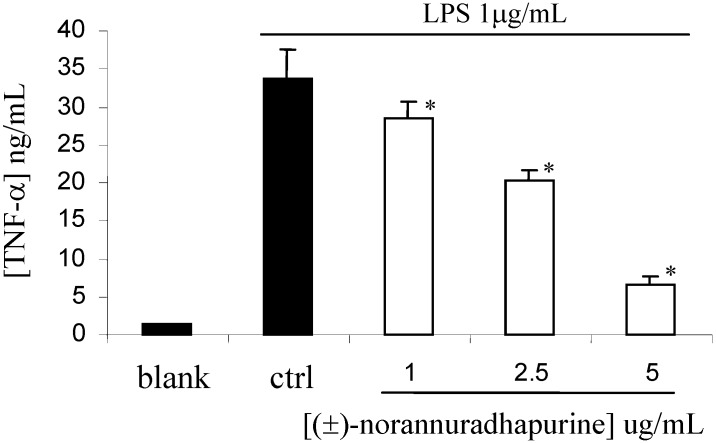
Effect of (±)-±)-norannuradhapurine on LPS-induced TNF-a production by RAW 264.7 cells. The values are the means of at least three determinations ± SD. Probability level (Student’s *t*-test): * *p* < 0.05 *vs*. LPS-treated group.

**Figure 6:**
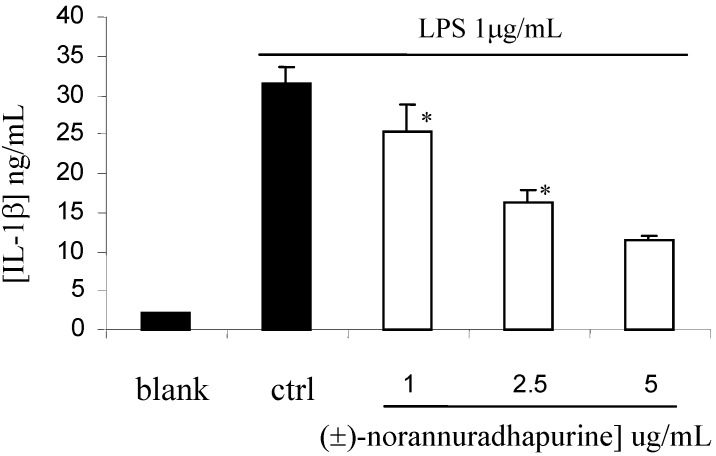
Effect of (±)-norannuradhapurine on IL1-β production by RAW 264.7 cells. The values are the means of at least three determinations± SD. Probability level (Student’s *t*-test): * *p* < 0.05 *vs*. LPS-treated group.

**Figure 7:**
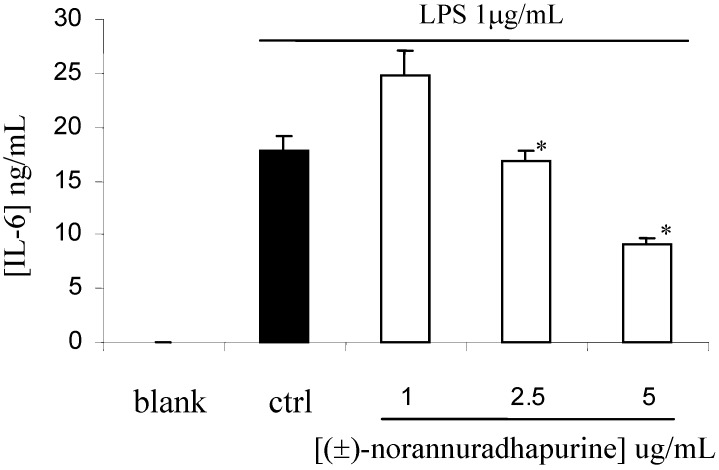
Effect of (±)-norannuradhapurine on LPS induced IL-6 production by RAW 264.7 cells. The values are the means of at least three determinations ± SD. Probability level (Student’s *t*-test): * *p* < 0.05 *vs*. LPS-treated group.

In contrast to iNOS and COX-2, (±)-norannuradhapurine had no effect on the expression of β-actin and COX-1 (data not shown). This finding indicates that (±)-norannuradhapurine could suppress NO and PGE_2_ production in LPS-stimulated RAW 264.7 cells by inhibiting iNOS and COX-2 protein expression, respectively. It has been reported that cytokines such as TNF-a, IL-1b and IL-6 are pro-inflammatory *in vitro* as well as *in vivo* [14]. The present study also demonstrated that (±)-norannuradhapurine has inhibitory effects on the production of TNF-a, IL-1b and IL-6 in LPS-stimulated RAW 264.7 cells. As shown in Figure 5, Figure 6 and Figure 7, LPS-induced productions of TNF-a, IL-1b and IL-6 were significantly inhibited by (±)-norannuradhapurine in a concentration-dependent manner. In addition, the cytotoxic effect of (±)-norannuradhapurine was evaluated in the absence or presence of LPS, (more than 95% cell viability). There is no significant difference on cell viability when treated with (±)-norannuradhapurine at all concentrations used (1-5 mg/mL) in the absence or presence of LPS. 

## Conclusions

In conclusion, we found that (±)-norannuradhapurine has anti-inflammatory activity, it inhibits the production of NO, PGE_2_, TNF-a, IL-1b and IL-6 and the expression of iNOS and COX-2 in macrophages stimulated with LPS *in vitro*. From these results it is expected that (±)-norannuradhapurine could be potentially useful for the treatment inflammatory diseases.

## Experimental

### General

Melting points were determined on a Stuart SMP2 apparatus and are uncorrected. Ultraviolet spectra were recorded on methanol solutions with a Jasco V-530 UV-VIS spectrophotometer. Infrared spectra were recorded on Nujol mulls unless stated otherwise with a Perkin-Elmer Spectrum GX FT-IR spectrophotometer. ^1^H- and ^13^C-NMR magnetic resonance spectra were recorded on CDCl_3_ solutions, unless stated otherwise, at 300 MHz for ^1^H and 75 MHz for ^13^C with a Bruker AVANCE 300 spectrometer. Tetramethylsilane was used as the internal standard. Mass spectra were measured on a Hewlett Packard 5989B spectrometer. Elemental microanalyses were performed with a Perkin-Elmer 2400 elemental analyser. 

*2-Benzyloxy-6-bromo-3-methoxybenzaldehyde* (**3a**). Using standard conditions, **3a** was obtained in 92.4% yield as a green solid, m.p.53-54°C (Lit. [11] m.p. 49 °C); ^1^H-NMR: d 10.23 (1H, s, CHO), 7.45-7.32 (6H, m, Ar-H), 6.96 (1H, d, *J* = 9.00 Hz, Ar-H), 5.10 (2H, s, PhCH_2_), 3.89 (3H, s, OCH_3_); ^13^C–NMR: d 190.4 (CH), 152.8 (C), 150.8 (C), 136.3 (C), 129.6 (CH), 129.1 (C), 128.7 (CH), 128.6 (CH), 128.5 (CH), 117.4 (CH), 112.4 (C), 76.5 (CH_2_), 56.2 (OCH_3_). 

*2-Benzyloxy-6-bromo-3-methoxybenzyl alcohol* (**3b**). Standard sodium borohydride reduction of **3a** gave **3b** in 94.6% yield as colourless prisms from ethanol, m.p. 84-85°C; Anal.Calc. for C_15_H_15_BrO_3_: C, 55.8; H, 4.7. Found: C, 55.6; H, 4.9%; ^1^H-NMR: d 7.45-7.29 (6H, m, Ar-H), 6.79 (1H, d, *J* = 9.0 Hz, Ar-H), 5.06 (2H, s, PhCH_2_), 4.72 (2H, d, *J* = 5.40 Hz, CH_2_), 3.87 (3H, s, OCH_3_), 2.14 (1H, br t, *J* = 5.40 Hz, OH); ^13^C–NMR: d 152.3 (C), 147.3 (C), 137.0 (C), 134.2 (C), 128.6 (CH), 128.5 (CH), 128.4 (CH), 128.1 (CH), 115.0 (C), 113.3 (CH), 75.8 (CH_2_), 60.4 (CH_2_), 56.0 (OCH_3_). 

*2-Benzyloxy-6-bromo-3-methoxybenzyl chloride* (**3c**). Treatment of **3b** with thionyl chloride gave crude **3c** as a white solid in 98.2% yield, m.p. 65-66°C. This was used in the next step without further purification. ^1^H-NMR: d 7.53-7.24 (6H, m, Ar-H), 6.82 (1H, d, *J* = 9.00 Hz, Ar-H), 5.11 (2H, s, PhCH_2_), 4.77 (2H, s, CH_2_), 3.87 (3H, s, OCH_3_); ^13^C–NMR: d 152.4 (C), 147.6 (C), 137.1 (C), 131.7 (C), 128.5 (CH), 128.4 (CH), 128.3 (CH), 128.2 (CH), 115.4 (C), 114.0 (CH), 75.6 (CH_2_), 56.1 (OCH_3_), 41.0 (CH_2_).

*2-Benzyloxy-6-bromo-3-methoxybenzyl cyanide* (**3d**)*.* Treatment of **3c** with sodium cyanide in acetone/water gave **3d** as colourless needles in 94.9% yield, m.p. 54-55°C; EI-MS (70 eV) *m/z* 333 [(M+2)^+^, 13%], 331 (M^+^, 14), 91(100); Anal. calc. for C_16_H_14_BrNO_2_: C, 57.9; H, 4.3; N, 4.2. Found: C, 57.7; H, 4.5; N, 4.0%; ^1^H-NMR: d 7.48-7.28 (6H, m, Ar-H), 6.83 (1H, d, *J* = 8.70 Hz, Ar-H), 5.12 (2H, s, PhCH_2_), 3.88 (3H, s, OCH_3_), 3.75 (2H, s, CH_2_); ^13^C-NMR: d 152.3 (C), 146.9 (C), 136.7 (C), 128.7 (CH), 128.6 (CH), 128.5 (CH), 128.0 (CH), 125.1 (C), 117.1 (C), 114.7 (C), 113.8 (CH), 75.3 (CH_2_), 56.1 (OCH_3_), 19.0 (CH_2_).

*2-Benzyloxy-6-bromo-3-methoxyphenylacetic acid* (**3e**). Hydrolysis of **3d** with potassium hydroxide in ethanol/water gave **3e** as colourless needles in 88.7% yield, m.p. 99-100°C; EI-MS (70 eV) *m/z* 352 [(M+2)^+^, 14%], 350 (M^+^, 14), 270(84), 244(61), 91(100). Anal. calc. for C_16_H_15_BrO_4_: C, 54.7; H, 4.3. Found: C, 54.9; H, 4.1%; ^1^H-NMR: d 7.44-7.26 (6H, m, Ar-H), 6.79 (1H, d, *J* = 9.00 Hz, Ar-H), 5.02 (2H, s, PhCH_2_), 3.87 (3H, s, OCH_3_), 3.84 (2H, s, CH_2_); ^13^C–NMR: d 175.9 (C), 152.1 (C), 147.4 (C), 137.1 (C), 128.8 (C), 128.5 (CH), 128.3 (CH), 128.2 (CH), 127.6 (CH), 116.0 (C), 112.9 (CH), 75.1 (CH_2_), 56.0 (OCH_3_), 35.8 (CH_2_). 

*2-(2-Benzyloxy-6-bromo-3-methoxyphenyl)-N-(3,4-methylenedioxyphenethyl)acetamide* (**4**). A mixture of 2-benzyloxy-6-bromo-3-methoxyphenylacetic acid (**3e**, 21.5 g) and thionyl chloride (20.3 g) in benzene (150 mL) was refluxed for 1 h, then the solvent and excess thionyl chloride were removed *in vacuo*. The resulting crude 2-benzyloxy-6-bromo-3-methoxyphenylacetyl chloride (**3f**) was dissolved in chloroform (200 mL) and added portionwise to a mixture of 2-(3,4-methylene-dioxyphenyl)ethylamine (**2**, 11.2 g) [9] in chloroform (100 mL), sodium hydrogen carbonate (25 g) and ice (200 g). The mixture was stirred at room temperature for 3 h. The chloroform layer was washed with 10% sodium carbonate (3 x100 mL), water (2 x100 mL), 5% HCl (3 x 100 mL), brine and then dried. Removal of the solvent under vacuum gave a pale yellow solid which was recrystallized from ethanol to give amide **4** as a pale yellow solid (21.6 g , 70.8%), m.p. 143-144°C; UV λ_max_ nm (MeOH) (log ε ) 231sh (4.09), 286 (3.77); IR ν_max_ (KBr): 3290 (NH), 3062, 3031, 3007, 2938, 2888, 2839, 1647 (C=O), 1550, 1504, 1489, 1471, 1441, 1406, 1366, 1344, 1274, 1249, 1129, 1073, 1040, 978, 927, 861, 805, 750, 696 cm^-1^; EI-MS(70 eV) *m/z* 499 [(M+2)^+^, 4%], 497 (M^+^, 4), 418 (10), 352(7), 270(9), 238(30), 223(14), 164(8), 148(82), 135(19), 91(100). Anal. calc. for C_25_H_24_BrNO_5_: C, 60.3; H, 4.9; N, 2.8. Found: C, 60.5; H, 4.7; N, 2.7%; ^1^H-NMR: d 7.46-7.23 (6H, m, Ar-H), 6.78 (1H, d, *J* = 8.70 Hz, Ar-H), 6.61 (1H, d, *J* = 7.80 Hz, Ar-H), 6.54 (1H, d, *J* = 1.50 Hz, Ar-H), 6.47 (1H, dd, *J* = 7.80, 1.50 Hz, Ar-H), 5.49 (1H, br s, N-H), 4.98 (2H, s, PhCH_2_), 3.88 (3H, s, OCH_3_), 3.68 (2H, s, CH_2_), 3.34 (2H, apparent q, *J* = 6.60 Hz, CH_2_N), 2.60 (2H, t, *J* = 6.60 Hz, CH_2_); ^13^C–NMR: d 169.3 (C), 152.3 (C) , 147.6 (C), 147.0 (C), 146.0 (C), 137.0 (C), 132.5 (C), 129.7 (C), 128.5 (C), 128.4 (CH), 128.3 (CH), 128.1 (CH), 121.6 (CH), 113.5 (CH), 112.7 (CH), 109.0 (CH), 108.2 (CH), 100.8 (CH_2_), 75.1 (CH_2_), 55.9 (OCH_3_), 40.8 (CH_2_), 38.4 (CH_2_), 35.2 (CH_2_). 

*1-(2-Benzyloxy-6-bromo-3-methoxybenzyl)-6,7-methylenedioxy-3, 4-dihydroisoquinoline* (**5**). A solution of amide **4** (6.03 g) and phosphorus oxychloride (19.4 g) in benzene (50 mL) was refluxed for 2 h. The excess reagent and solvent were removed under vacuum. The resulting brown residue was shaken with chloroform (80 mL) and dilute ammonium hydroxide (60 mL). The chloroform layer was washed with water (3 x 60 mL) and then dried. Removal of the solvent under vacuum followed by recrystallisation from ethanol gave dihydroisoquinoline **5** as pale yellow prisms (5.64 g, 97.1%), m.p. 81-82°C; UV λ_max_ nm (log ε) 227sh (4.52), 280 (3.91), 313 (3.90); IR ν_max_(KBr): 1636, 1600, 1574, 1266, 1233, 1173, 1129, 1097, 1076, 1038, 1013, 980, 934, 865, 798, 751, 697 cm^-1^; Anal. calc. for C_25_H_22_BrNO_4_: C, 62.5; H, 4.6; N, 2.9. Found: C, 62.7; H, 4.4; N, 2.8%; ^1^H-NMR: d 7.40-7.26 (6H, m, Ar-H), 7.25 (1H, s, Ar-H), 6.75 (1H, d, *J* = 8.70 Hz, Ar-H), 6.63 (1H, s, Ar-H), 5.00 (2H, s, PhCH_2_), 4.17 (2H, s, CH_2_), 3.85 (3H, s, OCH_3_), 3.50 (2H, t, *J* = 7.20 Hz, CH_2_), 2.46 (2H, t, *J* = 7.20 Hz, CH_2_); ^13^C–NMR: d 163.8 (C), 152.2 (C), 148.8 (C), 147.4 (C), 146.3 (C), 137.7 (C), 133.3 (C), 132.6 (C), 128.3 (CH), 128.1 (CH), 127.8 (CH), 127.7 (CH), 123.5 (C), 116.3 (C), 112.0 (CH), 107.8 (CH), 105.6 (CH), 101.2 (CH_2_), 74.8 (CH_2_), 55.8 (OCH_3_), 46.8 (CH_2_), 37.0 (CH_2_), 26.3 (CH_2_). 

*1-(2-Benzyloxy-6-bromo-3-methoxybenzyl)-6,7-methylenedioxy-1,2,3,4-tetrahydroisoquinoline* (**6a**). Sodium borohydride (2.0 g) was added portionwise to a stirred solution of dihydroisoquinoline **5** (18.2 g) in ethanol (310 mL) and the mixture was refluxed for 1 h. Chloroform (200 mL) was added and the mixture was washed with water (4 x 200 mL), brine and then dried. Removal of the solvent under vacuum gave tetrahydroisoquinoline **6a** as a yellow-red viscous oil (14.8 g, 81.0%) which failed to crystallise and was used in the next step without further purification. ^1^H-NMR: d 7.47-7.26 (6H, m, Ar-H), 6.73 (1H, d, *J* = 8.70 Hz, Ar-H), 6.66 (1H, s, Ar-H), 6.50 (1H, s, Ar-H), 5.84 (2H, s, OCH_2_O), 5.05 and 4.92 (each 1H, d, *J* = 10.80 Hz, PhCH_2_), 4.23 (1H, dd, *J* = 10.50, 2.70 Hz, H-1), 3.85 (3H, s, OCH_3_), 3.30-2-43 (6H, m, CH_2_); ^13^C-NMR: d 152.1 (C), 147.6 (C), 145.8 (C), 145.6 (C), 137.3 (C), 133.5 (C), 131.9 (C), 128.5 (CH), 128.3 (CH), 128.2 (CH), 128.0 (CH), 127.6 (C), 115.9 (C), 112.0 (CH), 108.5 (CH), 106.9 (CH) 100.5 (CH_2_), 75.0 (CH_2_), 55.8 (OCH_3_), 55.5 (CH), 39.4 (CH_2_), 37.6 (CH_2_), 29.9 (CH_2_).

*2-Trifluoroacetyl-1-(2-benzyloxy-6-bromo-3-methoxybenzyl)-6,7-methylenedioxy-1,2,3,4-tetrahydro-isoquinoline* (**6b**). Trifluoroacetic anhydride (38.5 g) was added dropwise to a stirred mixture of tetra-hydroisoquinoline **6a** (14.8 g) and triethylamine (26.6 g) in chloroform (230 mL) at 0-10°C. Stirring was continued at room temperature for 3 h. Chloroform (150 mL) was added and the chloroform layer was washed with 10% sodium hydrogen carbonate (4 x 200 mL), 10% HCl (3 x 250 mL), brine and then dried. Removal of the solvent gave a red-brown viscous oil which crystallized from ethanol to give trifluoroacetamide **6b** as a pale yellow solid (7.0 g, 39.4%); m.p. 122-123°C; UV λ_max_ nm (MeOH) (log ε ) 231sh (4.17), 289 (3.80); IR ν_max_ (KBr): 1681, 1571, 1504, 1300, 1284, 1269, 1235, 1190, 1180, 1158, 1082, 1036, 984, 976, 961, 941, 912, 855, 795, 759, 699 cm^-1^; ^1^H-NMR (*d_6_*-DMSO): d 7.47-7.31 (5H, m, Ar-H), 7.23 (1H, d, *J* = 8.70 Hz, Ar-H), 6.82 (1H, d, *J* = 8.70 Hz, Ar-H), 6.54 (1H, s, Ar-H), 6.37 (1H, s, Ar-H), 5.91 (2H, s, OCH_2_O), 5.68 (1H, dd, *J* = 10.70, 3.20 Hz, H-6a), 5.16 and 4.95 (each 1H, d, *J* = 10.80 Hz, PhCH_2_), 3.88 (3H, s, OCH_3_), 3.85-3.65 (1H, m, CH), 3.30-2.54 (5H, m, CH_2_); ^13^C–NMR: (*d_6_*-DMSO): d 155.1 (C), 151.6 (C), 147.8 (C), 146.6 (C), 146.2 (C), 137.2 (C), 130.7 (C), 128.4 (CH), 128.3 (CH), 128.2 (CH), 128.1 (CH), 127.1 (C), 125.8 (C), 118.2 (C), 115.5 (C), 113.0 (CH), 108.0 (CH), 106.6 (CH), 100.8 (CH_2_), 74.7 (CH_2_), 55.9 (OCH_3_), 53.4 (CH), 39.8 (CH_2_), 36.6 (CH_2_), 29.0 (CH_2_). 

*8-Benzyloxy-9-methoxy-1,2-methylenedioxy-6-trifluoroacetylnoraporphine* (**1b**). A solution of 2,2'-azobis(isobutyronitrile) (1.59 g) and tributyltin hydride (6.18 g) in toluene (70 mL) was added in 6 equal portions over 2.5 h to a refluxing solution of trifluoroacetamide **6b** (6.23 g) in toluene (110 mL). The resulting mixture was then refluxed for 24 h. The solvent was removed under vacuum and the resulting yellow residue was dissolved in acetonitrile (500 mL) and washed with hexane (5 x 200 mL) and then dried. Removal of the solvent under vacuum gave a yellow solid which was triturated from ethanol to give noraporphine **1b** as colourless needles (2.15 g, 40.1%); m.p. 218 -219°C; UV λ_max_ nm (log ε ) 282 (4.33), 323sh (3.71); IR ν_max_ (KBr): 1681 (C=O), 1601, 1573, 1273, 1245, 1236, 1215, 1202, 1187, 1173, 1153, 1136, 1080, 1061, 1037, 944, 919, 847, 816, 735, 694, 651 cm^-1^; EI-MS (70 eV) m/z 497(M^+^, 91%), 406(91), 374(45), 309(24), 91(100). Anal. calc. for C_27_H_22_F_3_NO_5_: C, 65.2; H, 4.5; N, 2.8. Found: C, 65.0; H, 4.7; N, 3.0%; ^1^H-NMR: d7.86 (1H, d, *J* = 8.70 Hz, H-11), 7.60-7.25 (5H, m, Ar-H), 6.91 (1H, d, *J* = 8.70 Hz, H-10), 6.54 (1H, s, H-3), 6.02 (2H, AB q, *J* = 1.20 Hz, OCH_2_O), 5.10 and 4.97 (each 1H, d, *J* = 10.80 Hz, PhCH_2_), 4.94-4.83 (1H, m, H-6a), 4.18 (1H, br d, *J* = 13.20 Hz, Ha-5), 3.91 (3H, s, OCH_3_), 3.63-2.35 (5H, m, CH_2_); ^13^C–NMR: d 155.8 (C), 152.6 (C), 147.1 (C), 145.2 (C), 142.8 (C), 137.6 (C), 129.6 (C), 128.6 (CH), 128.4 (CH), 128.0 (CH), 126.4 (C), 123.8 (C), 123.6 (CH), 118.3 (C), 117.5 (C), 114.5 (C), 110.6 (CH), 106.7 (CH), 101.0 (CH_2_), 75.1 (CH_2_), 55.8 (OCH_3_), 52.3 (CH), 41.4 (CH_2_), 30.4 (CH_2_), 26.8 (CH_2_).

*8-Hydroxy-9-methoxy-1, 2-methylenedioxy-6-trifluoroacetylnoraporphine* (**1c**). A solution of noraporphine **1b** (2.0 g) in methanol (100 mL) and chloroform (250 mL) was hydrogenolysed in the presence of 10% Pd/C (2.8 g) at 45-50 psi for 4 h. The catalyst was filtered off and the solvent removed under vacuum. The resulting white residue was shaken with chloroform (150 mL) and 10% ammonium hydroxide (100 mL). Removal of the solvent under vacuum followed by recrystallisation form ethanol gave noraporphine **1c** as pale brown needles (0.65 g , 40.0%); m.p. 287-289 °C; UV λ_max_ nm (log ε ) 284 (4.24), 298 (4.12), 321sh (3.81); IR ν_max_ (KBr): 3459 (OH), 1674 (C=O), 1613, 1583, 1275, 1240, 1196, 1186, 1175, 1161, 1145, 1078, 1061, 1023, 940, 916, 897, 845, 810, 764, 757 cm^-1^; EI-MS(70 eV) *m/z* 407(M^+^,74%), 376(13), 346(30), 327(20), 305(64), 281(100), 272(39), 256(39), 206(28), 167(30), 91(100); Anal. calc. for C_20_H_16_F_3_NO_5_: C, 59.0; H, 4.0; N, 3.4. Found: C, 59.2; H, 3.8; N, 3.2%; ^1^H-NMR: *d* 7.69 (1H, d, *J* = 8.70 Hz, H-11), 6.87 (1H, d, *J* = 8.70 Hz, H-10), 6.59 (1H, s, H-3), 6.06 (2H, AB q, *J* = 1.20 Hz, OCH_2_O), 5.13 (1H, dd, *J* = 13.50, 4.50 Hz, H-6a), 4.30-4.18 (1H, m, Ha-5), 3.96 (3H, s, OCH_3_), 3.66-2.48 (5H, m, CH_2_); ^13^C-NMR (*d_6_*-DMSO): d 154.6 (C), 146.9 (C), 146.0 (C), 142.6 (C), 141.4 (C), 127.8 (C), 126.8 (C), 124.1 (C), 122.4 (C), 118.1 (CH), 116.1(C), 109.0 (CH), 106.8 (CH), 100.1 (CH_2_), 55.6 (OCH_3_), 53.0 (CH), 43.0 (CH_2_), 29.1 (CH_2_), 29.0 (CH_2_). 

*(±)-Norannuradhapurine* (**1a**). Noraporphine **1c** (0.77 g) was dissolved in methanol (140 mL) with heating. Potassium carbonate (1.46 g) in water (4.5 mL) was added and then the mixture was refluxed for 2.5 h. The solvent was removed under vacuum. The resulting residue was shaken with water (60 mL) and 10% sodium carbonate (40 mL), and extracted with chloroform (4 x 40 mL) and then dried. Removal of the solvent under vacuum followed by recrystallisation form ethanol gave (±)norannuradhapurine (**1a**) as purple needles (0.46 g, 78.2%) ; m.p. 207-209°C; UV λ_max_ nm (log ε ) 217 (4.43), 283 (4.24), 298sh (4.09), 320sh (3.76); IR ν_max_ (KBr): 3276 (OH), 2583, 1608, 1578, 1288, 1258, 1235, 1167, 1146, 1127, 1083, 1059, 1023, 991, 947, 909, 841, 792 cm^-1^; EI-MS(70 eV) *m/z* 311(M^+^, 54%), 310 (100), 278(17), 91(14); Anal. calc. for C_18_H_17_NO_4_: C, 69.4; H, 5.5; N, 4.5. Found: C, 69.6; H, 5.3; N, 4.6%; ^1^H-NMR: d 7.64 (1H, d, *J* = 8.40 Hz, H-11), 6.82 (1H, d, *J* = 8.40 Hz, H-10), 6.54 (1H, s, H-3), 6.00 (1H, AB q, *J* = 1.50 Hz, 2H, OCH_2_O), 3.92 (3H, s, OCH_3_), 3.88 (1H, dd, *J* = 13.80, 5.10 Hz, H-6a), 3.45-2.34 (6H, m, CH_2_); ^13^C-NMR (CDCl_3_): d 146.5 (C), 145.9 (C), 142.2 (C), 142.0 (C), 127.8 (C), 126.8 (C), 125.0 (C), 121.5 (C), 118.8 (CH), 116.4 (C), 108.4 (CH), 107.3 (CH), 100.5 (CH_2_), 56.0 (OCH_3_), 53.3 (CH), 43.5 (CH_2_), 29.6 (CH_2_), 29.2 (CH_2_); ^13^C NMR (*d_6_*-DMSO) d/ppm 147.0 (C), 145.9 (C), 142.7 (C), 141.4 (C), 128.0 (C), 126.9 (C), 124.2 (C), 122.6 (C), 118.1 (CH), 116.1 (C), 109.4 (CH) , 106.8 (CH), 100.1 (CH_2_), 55.8 (OCH_3_), 53.0 (CH), 43.0 (CH_2_), 29.2 (CH_2_), 29.1 (CH_2_). 

### Minimum inhibitory concentration (MIC)

MIC of (±)-norannuradhapurine was determined by NCCLS microbroth dilution methods [12]. (±)-norannuradhapurine was weighed and dissolved in DMSO to make a solution of concentration 2.56 mg/mL. From this stock solution two-flow serial dilution has been carried out to give a series of solutions from 256 mg/mL to 0.50 mg/mL with culture medium in 96-well microplates (100 μl of total volume). Three different microorganisms were selected *viz. Staphytolcoccus aureus* ATCC25932, *Escherichia coli* ATCC10536 and *Candida albicans* ATCC90028. They were subcultured on nutrient broth supplemented with 10% glucose (NBG) (for bacteria) or Sabouraud glucose broth (for yeast) and incubated at 37 °C for 24 h. A final concentration of 1 x 10^5^ cfu/mL of test bacteria or yeast was added to each dilution. The plates were incubated at 37 °C for 48 h. MIC was defined as the lowest concentration of test agent that inhibited bacterial or yeast growth, as indicated by the absence of turbidity. Test agent-free broth containing 5% DMSO was incubated as growth control. 

### Anti-inflammatory activity

Murine macrophage RAW 264.7 cell line obtained from American Type Culture Collection (ATCC, Maryland, USA), was maintained in DMEM supplemented with 10% heat inactivated FBS, penicillin G (100 IU/mL), streptomycin (100 mg/mL), and L-glutamine (2 mM) and incubated at 37 °C in a humidified atmosphere containing 5% CO_2_. Cells (1 x 10^6^ /mL) were pre-incubated 2 h with (±)-norannuradhapurine (1, 2.5 and 5 mg/mL) and further cultured 24 h with LPS (1 mg/mL) in 24-well plates. Supernatants were removed at the allotted times and NO, PGE_2_, TNF-a, IL-1b and IL-6 levels were quantified by immunoassay kits according to the manufacture’s protocols (Assay Designs’ Correlate-EIA^TM^, Stressgen, USA), respectively. Western blot Cellular proteins were extracted from control and (±)-norannuradhapurine-treated RAW 264.7 cells. The washed cell pellets were resuspended in lysis buffer buffer (50 mM HEPES pH 7.0, 250 mM NaCl, 5 mM EDTA, 0.1% Nonidet P-40, 1 mM phenylmethylsulfonyl fluoride, 0.5 mM dithiothreitol, 5 mM NaF, 0.5 mM Na orthovanadate) containing 5 mg/mL each of leupeptin and aprotinin and incubated for 15 min at 4 °C. Cell debris was removed by microcentrifugation, followed by quick freezing of the supernatants. Protein concentration was determined by BioRad protein assay reagent according to the manufactures instruction, 40-50 mg of cellular proteins from treated and untreated cell extracts were electroblotted onto nitrocellulose membrane following separation on a 10% SDS-polyacrylamide gel electrophoresis. The immunoblot was incubated overnight with blocking solution (5% skim milk) at 4 °C, followed by incubation for 4 h with a 1:500 dilution of monoclonal anti-iNOS and COX-2 antibody (Santacruz, CA, USA). Blots were washed 2 times with PBS and incubated with a 1:1000 dilution of horseradish peroxidase-conjugated goat anti-mouse IgG secondary antibody (Santacruz, CA, USA) for 1 h at room temperature. Blots were again washed three times in Tween 20/Tris-buffered saline (TTBS) and then developed by enhanced chemiluminescence (Amersham Life Science, Arlington Heights, IL, USA). 

Cytotoxicity assay. 3-(4,5-Dimethylthiazol-2-yl)-2,5-diphenyltetrazolium bromide (MTT) cytotoxicity assay was performed according to the method previously described [13]. MTT solution was added at a concentration of 50 mg/mL into each well, which also contain 1, 2.5 and 5 mg/mL of (+)-3-methoxynordomesticine. After 4 h of incubation at 37 °C, the medium was discarded and the formazan blue, which formed in the cells, was dissolved in 50 μl DMSO. The optical density at 540 nm was determined with a microplate reader. The optical density of formazan formed in control (untreated) cells was taken as 100% of viability. 
